# The Aldosterone Blockade for Health Improvement Evaluation in End-Stage Renal Disease (ACHIEVE) Trial: Rationale and Clinical Research Protocol

**DOI:** 10.1177/20543581251348187

**Published:** 2025-06-03

**Authors:** Michael Walsh, David Collister, Martin Gallagher, Patrick B. Mark, Janak R. de Zoysa, Jessica Tyrwhitt, Karthik Tennankore, Laura Sola, Gilmar Reis, Denis Xavier, Russell Villanueva, Wen J. Liu, Camilo Félix, Li Zuo, Mustafa Arici, Vivekanand Jha, Ron Wald, Amanda Y. Wang, Atiya R. Faruqui, Fei Yuan, Shun Fu Lee, Alena Kuptsova, Courtney Christou, P. J. Devereaux, For the ACHIEVE Investigators

**Affiliations:** 1 Population Health Research Institute, Hamilton Health Sciences, McMaster University, Hamilton, ON, Canada; 2 Departments of Medicine and Health Research Methods, Evidence and Impact, McMaster University, Hamilton, ON, Canada; 3 St. Joseph’s Hospital, Hamilton, ON, Canada; 4 Department of Medicine, University of Alberta, Edmonton, AB, Canada; 5 South West Sydney Clinical Campus, UNSW, Liverpool, NSW, Australia; 6 The George Institute for Global Health, UNSW, Sydney, NSW, Australia; 7 School of Cardiovascular and Metabolic Health, University of Glasgow, UK; 8 University of Auckland, New Zealand; 9 Renal Services, Waitematā Health New Zealand, Auckland, New Zealand; 10 Division of Nephrology, Department of Medicine, Dalhousie University, Halifax, NS, Canada; 11 Nova Scotia Health, Halifax, NS, Canada; 12 Dialysis Unit, CASMU-IAMPP, Montevideo, Uruguay; 13 Pontificia Universidade Católica de Minas Gerais, Belo Horizonte, Brazil; 14 St. John’s Medical College and Research Institute, Bangalore, India; 15 Philippine General Hospital, Manila, Philippines; 16 Department of Nephrology, Hospital Sultanah Aminah, Johor Bahru, Malaysia; 17 Grupo de Investigación CENIEC, Facultad de Ciencias de la Salud Eugenio Espejo, Universidad UTE, Quito, Ecuador; 18 Department of Nephrology, Peking University People’s Hospital, Beijing, China; 19 Faculty of Medicine, Hacettepe University, Ankara, Türkiye; 20 George Institute for Global Health, University of New South Wales, New Delhi, India; 21 School of Public Health, Imperial College, London, UK; 22 Prasanna School of Public Health, Manipal Academy of Higher Education, India; 23 Division of Nephrology, St. Michael’s Hospital, Toronto, ON, Canada; 24 Department of Nephrology and Hypertension, Tel Aviv Medical Center, Israel; 25 Faculty of Medicine and Health Sciences, Macquarie University, Sydney, NSW, Australia; 26 Department of Renal Medicine, Concord Repatriation General Hospital, Concord Clinical School, University of Sydney, NSW, Australia

**Keywords:** kidney failure, dialysis, spironolactone

## Abstract

**Background:**

The mineralocorticoid aldosterone may contribute to the risk of cardiovascular morbidity and mortality in patients receiving maintenance dialysis. Whether spironolactone, a mineralocorticoid receptor antagonist, improves outcomes for patients receiving maintenance dialysis is unclear.

**Objective:**

To assess the efficacy and safety of spironolactone in patients receiving maintenance dialysis.

**Design:**

Placebo-controlled, randomized controlled trial.

**Setting:**

Dialysis units

**Patients:**

Patients receiving maintenance dialysis who are adherent to and able to tolerate spironolactone 25 mg daily during an open-label run-in period of at least 49 days were randomized to spironolactone 25 mg daily or matching placebo.

**Measurements:**

Randomized participants were followed for the primary outcome of cardiovascular death or hospitalization due to heart failure. Secondary outcomes include cause specific deaths, hospitalization due to heart failure, all-cause death, all-cause hospitalizations, and severe hyperkalemia. All deaths and possible hospitalizations for heart failure were adjudicated.

**Methods:**

Eligible participants received open-label spironolactone 25 mg daily for at least 7 weeks during a run-in period. Participants who tolerated and adhered to treatment were randomly allocated to continue spironolactone 25 mg daily or a matching placebo. We followed participants until trial close.

**Results:**

The trial began recruitment in 2018 and concluded recruitment in December 2024. Despite a reduced rate of recruitment during the global COVID-19 pandemic 3565 eligible participants were enrolled of whom 2538 were randomized to spironolactone or placebo from 143 dialysis programs.

**Limitations:**

Limited funding and the trial was stopped early due to futility to demonstrate an effect.

**Conclusions:**

ACHIEVE was designed as a large, simple trial to determine if spironolactone 25 mg daily prevents cardiovascular mortality and heart failure hospitalizations in patients with kidney failure receiving maintenance dialysis. ACHIEVE demonstrates the possibility of conducting large, international, investigator initiated randomized controlled trials for patients with kidney failure receiving dialysis.

NCT03020303.

## Abrégé

**Contexte:**

L’aldostérone, un minéralocorticoïde, pourrait avoir une incidence sur le risque de morbidité et de mortalité cardiovasculaires chez les patients sous dialyse d’entretien. L’efficacité de la spironolactone, un antagoniste des récepteurs des minéralocorticoïdes, pour améliorer les résultats dans cette population de patients reste à déterminer.

**Objectif:**

Évaluer l’efficacité et l’innocuité de la spironolactone chez les patients sous dialyse d’entretien.

**Conception:**

Essai randomisé contrôlé par placebo.

**Cadre:**

Unités de dialyse

**Sujets:**

Des patients sous dialyse d’entretien ont été randomisés pour recevoir une dose quotidienne de spironolactone 25 mg ou un placebo correspondant, à la suite d’un essai ouvert de pré-inclusion. Ont été inclus les sujets qui avaient pris et toléré une dose quotidienne de spironolactone (25 mg/jour) pendant au moins 49 jours durant l’essai de pré-inclusion.

**Mesures:**

Les sujets randomisés ont été suivis pour le critère de jugement principal, soit le décès de cause cardiovasculaire ou une hospitalisation pour insuffisance cardiaque. Les résultats secondaires comprennent les décès liés de cause spécifique, les hospitalisations pour insuffisance cardiaque, les décès et hospitalisations toutes causes confondues et l’hyperkaliémie grave. Tous les décès et hospitalisations liés à une possible insuffisance cardiaque ont été révisés.

**Méthodologie:**

Les sujets admissibles ont reçu 25 mg de spironolactone par jour en ouvert pendant au moins sept semaines (période de pré-inclusion). Les sujets qui ont pris et toléré le traitement pendant la pré-inclusion ont été randomisés pour poursuivre le traitement (spironolactone 25mg/jour) ou recevoir un placebo correspondant. Les sujets ont été suivis jusqu’à la clôture de l’essai.

**Résultats:**

Le recrutement des sujets a débuté en 2018 et s’est terminé en décembre 2024. Malgré une réduction du taux de recrutement pendant la pandémie de COVID-19, un total de 3565 patients admissibles ont été recrutés, desquels 2538 ont été randomisés pour recevoir la spironolactone ou un placebo. Les sujets participant à l’essai sont issus de 143 programmes de dialyse.

**Limites:**

Financement limité. Essai arrêté tôt en raison de la futilité de démontrer un effet.

**Conclusion:**

L’essai ACHIEVE, qui a été conçu comme un essai simple de vaste envergure, visait à déterminer l’efficacité d’une dose quotidienne de 25 mg de spironolactone pour prévenir la mortalité cardiovasculaire et les hospitalisations pour insuffisance cardiaque chez les patients atteints d’insuffisance rénale recevant une dialyse d’entretien. L’essai ACHIEVE démontre la possibilité de mener, à l’initiative des investigateurs, des essais contrôlés randomisés internationaux de grande envergure chez les patients dialysés.

## Introduction

Globally, approximately 3 million people receive dialysis for kidney failure.^1,2^ This number is increasing due to the epidemics of obesity, diabetes and vascular disease and as access to dialysis in low- and middle-income countries increases.^3^ Outcomes for patients who receive dialysis remain poor due to frequent hospitalizations, poor health-related quality of life, and high mortality rates.^2,4^ The importance of this is underscored by estimates that chronic kidney disease is the third fastest-rising cause of death according to the most recent Global Burden of Disease Study.^5^

Cardiovascular disease is the leading cause of death and hospitalization amongst dialysis recipients causing 8.9 deaths per 100 patient-years (42% of all deaths) and 55 hospitalizations per 100 patient-years.^2^ Observational studies suggest progressive ventricular dilatation and cardiac fibrosis with consequent heart failure as an important causal pathway for cardiovascular morbidity and mortality.^6,7^ Notably, few interventions have shown clear benefits on these outcomes.^8-10^

Aldosterone, a mineralocorticoid, is associated with myocardial fibrosis in animal models and with myocardial remodeling in humans.^11,12^ Aldosterone is frequently elevated in people with kidney failure receiving dialysis, and is associated with sudden death.^13-15^ Mineralocorticoid receptor antagonists (MRAs) reduces cardiovascular deaths and heart failure hospitalizations in patients with chronic kidney disease and patients with symptomatic heart failure without kidney failure.^16-20^ Randomized trials of MRAs in patients with kidney failure suggest they may be effective, but were too small/lacked sufficient events to be sufficiently certain and suggest potential harms from hyperkalemia.^21-23^

We are conducting an international randomized controlled trial (RCT) comparing spironolactone, the most widely available MRA, to placebo to determine if spironolactone reduces cardiovascular death and hospitalizations for heart failure in patients with kidney failure receiving dialysis. This RCT is called ***A* ldosterone blo *C* kade for *H* ealth *I* mprovement *EV* aluation in *E* nd-stage renal disease (ACHIEVE)**.

## Methods

**Figure 1.**
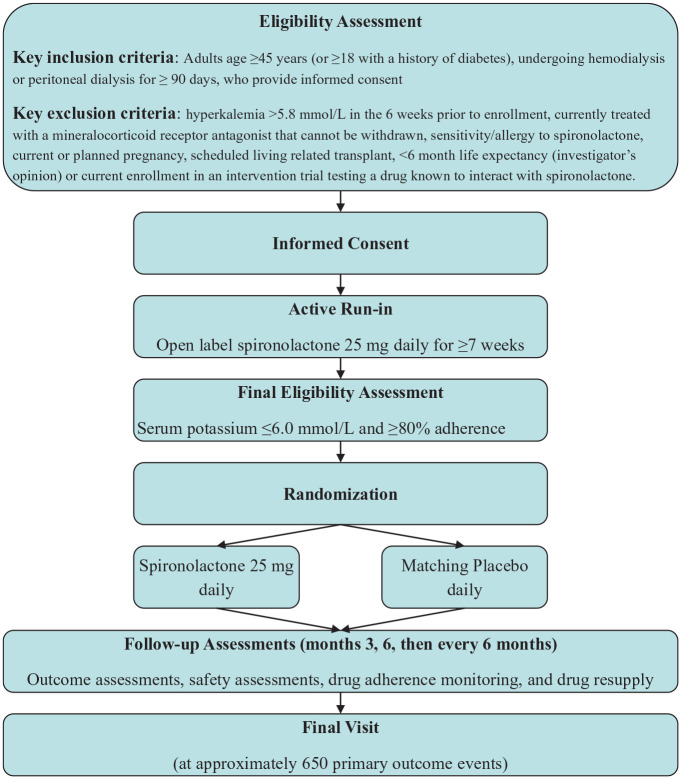
ACHIEVE study flowchart.

ACHIEVE is a multicenter, international, 2 parallel-arm RCT with an active run-in period among people undergoing maintenance dialysis. Following screening and consent, participants entered an open-label active run-in period in which they received spironolactone 25 mg daily by mouth for 7 to 14.5 weeks. If participants remained eligible after run-in, they were randomized to continue receiving spironolactone 25 mg daily or matching placebo (Figure 1). The 25 mg dose of spironolactone was chosen on the basis of its safety and suggestion of benefit in prior trials of patients receiving dialysis, and the increased half-life of canrenone, an active metabolite of spironolactone, by 1.5 to 2 times in kidney failure suggesting that the 25 mg dose was similar to the administered dose of 37.5 to 50 mg per day used in a pivotal heart failure trials and it is considered equivalent to eplerenone 50 mg daily which we previously demonstrated is safe for patients receiving dialysis and was effective at reducing mortality in heart failure.^17,21,24-28^

### Participant Eligibility

The eligibility criteria of the trial were designed to reflect the idea that most patients receiving dialysis, regardless of how long they had been receiving dialysis, were at risk of primary outcome. The criteria were therefore broadly inclusive save the exclusion of patients at high risk of hyperkalemia (for safety), nonadherence (to maximize the ability to detect treatment effects), or very near-term death or transplantation as there would be little chance to modify those patients’ outcomes. The inclusion and exclusion criteria are listed in Table 1.

**Table 1.** Eligibility Criteria for the ACHIEVE Trial.

Inclusion criteria (included if all criteria met):
≥45 years old or ≥18 old with a history of diabetes and;
Receiving dialysis ≥90 days and;
Receiving either hemodialysis (prescribed at least 2 treatments per week) or peritoneal dialysis (prescribed with at least 1 exchange daily) and;
Provide informed consent.
Exclusion criteria (exclude if any criteria met):
Hyperkalemic defined as a serum potassium >5.8 mmol/L in the 6 weeks prior to enrollment or a serum potassium >6.0 mmol/L during active run-in or;
Taking and unable to withdraw from a mineralocorticoid receptor antagonist or:
Known to have a sensitivity or allergy to spironolactone or;
Planning a pregnancy or breastfeeding or;
Scheduled for a living related donor renal transplant or;
Expected to live <6 months in the opinion of a treating nephrologist or;
Enrolled in another interventional trial testing a mineralocorticoid receptor antagonist or drug that has a known or likely interaction with spironolactone or;
Treating physician believes either spironolactone is either absolutely indicated or absolutely contra-indicated.

An active run-in period was utilized to enrich the randomized population for patients who would both take the study medication and could tolerate the study medication for at least 7 weeks and a maximum of 14.5 weeks. We chose a minimum duration of 7 weeks because no MRA induced hyperkalemia was seen after this in our pilot work and the maximum duration was based on the practical provision of 100 tables in the run-in medication supply and possibility of withdrawal effect from longer run-in durations.^27^ This run-in period reduced the intensity of monitoring required during the randomized phase since participants were already known to tolerate the drug without significant side effects. In addition, the active run-in phase limits the generalizability of the results to patients able to tolerate spironolactone. This better represents the target for intervention since those who cannot or do not take a medication would not derive benefit from that medication.

Potentially eligible patients were consented for the active run-in period and received open-label spironolactone 25 mg orally daily for at least 7 weeks to determine if spironolactone was safe and tolerated for chronic use prior to randomization. Spironolactone and matching placebo were purchased from Hangzhou Minsheng Pharmaceutical Co. for participants from China and from Tiofarma and Teva for the rest of the world. Placebos for Teva product were manufactured by a third party based on the Teva spironolactone specifications. Participants were assessed for hyperkalemia at 1, 2, 3, and 7 weeks. Those that were adherent, defined as having taken at least 80% of the study medication by self-assessment, without a serum potassium >6.0 mmol/L and willing to continue taking the study medication were randomly allocated to continue spironolactone 25 mg daily or a matching placebo. The adequacy of self-assessed adherence was examined in a substudy of our run-in participants and previously published.^29^ Random allocation was accomplished using a concealed, random number list prepared by an independent statistician with randomly permuted block sizes, stratified by center, using an interactive web-based randomization system.

### Follow-Up

Randomly allocated participants were followed every 6 months with an additional assessment at 3 months after randomization to reassess safety and reinforce adherence to the study medication. The duration of follow-up was until the center close out with a staggered close out starting approximately 1 year from the anticipated last event to accommodate varying financial and logistic constraints for individual sites. Follow-up assessments were designed to concentrate on ascertaining participant safety and major outcomes including all hospitalizations, encouraging participant adherence to the study medication, dispensation of study medication, and collecting additional outcomes of interest such as health-related quality of life. The use of potassium binders or exchange resins and changes in dialysate potassium or other medications were left to the discretion of the local treating physicians. Medication could be down-titrated to 3 times per week or temporarily discontinued if it was thought to be the cause of an adverse effect such as gynecomastia or severe hyperkalemia. When the local investigator felt it was safe to do so, the study medication could be restarted or uptitrated from 3 times per week to daily. Modifications to other medications or the dialysis prescription were left to the discretion of the local investigator and health care teams.

### Outcomes

The primary outcome of ACHIEVE was a composite of cardiovascular mortality or hospitalization due to heart failure analyzed as time to first event. All deaths and all potentially relevant hospitalizations were adjudicated by blinded outcome adjudicators. Any potential primary outcome that was refuted by the adjudicator was also reviewed by an adjudication co-chair as a “tie-breaker.” Outcome definitions are listed in Appendix 2. Secondary outcomes included components of the composite primary outcome, all-cause hospitalizations, all-cause deaths and severe hyperkalemia (Table 2).

**Table 2.** ACHIEVE Primary and Secondary Outcomes.

Primary
Time to first composite of cardiovascular death or hospitalization for heart failure
Secondary
Time to cause specific death (cardiac, vascular, noncardiovascular)
Time to first hospitalization for heart failure
All-cause death
Time to first all-cause hospitalization
Severe hyperkalemia

### Serious Adverse Events and Participant Safety

The safety profile of spironolactone is well-known so, as recommended by regulatory authorities, adverse events that led to discontinuation or dose modification of study drug were recorded. We also recorded all hospitalizations. Other serious adverse events were recorded if the investigator considered them related to study drug, but we exempted them from expedited reporting unless they were unexpected in terms of the study medication product monograph/summary of product characteristics list of side effects or the participant’s medical history and were not already being recorded as a study outcome. Our rationale for this approach was that serious events unrelated to the study medication and already known are unlikely to contribute significantly to the understanding of spironolactone’s potential harms when reported as isolated events in a blinded fashion. Instead, our external safety and efficacy monitoring committee reviewed unblinded information, including all hospitalizations and deaths to better determine if spironolactone was causing serious adverse events.

The External Safety and Efficacy Monitoring Committee met twice per year to review trial data with interim efficacy analyses scheduled at approximately 33% and 75% of the expected primary outcome events. They applied a modified Haybittle-Peto approach for interim analyses of 4 standard deviations for analyses in the first half of the trial and 3 in the second half. The alpha level for the final analyses is .049659 to correct for the type I error rate spent during the interim analyses.

### Sample Size Estimation

Our sample size estimates took into consideration the issues of the competing risk of noncardiovascular death, kidney transplantation, nonadherence to study medication, and losses to follow-up based on kidney failure populations in epidemiologic studies and other clinical trials.^30^

We made the following assumptions when we originally designed ACHIEVE and then conducted a blinded sample size re-estimation revising these assumptions in 2023 when it became clear recruitment was slower than anticipated due to a staged start-up phase and the effects of the global COVID-19 pandemic:

1. Effect size: We aimed to detect a hazard ratio of 0.75 for the primary outcome. Other studies of MRA in nondialysis patients that are not immediately post-MI, demonstrated hazard ratios from 0.63 to 0.82 for a similar outcome and our meta-analysis of MRA trials in dialysis suggested an RR 0.34 (95% CI 0.15 to 0.75).^8-10,17,27,28^ Our targeted effect size was therefore approximately the mid-point of nondialysis trials and the upper bound of the 95% CI of dialysis trials for mortality.
2. Control outcome rate: We estimated a primary outcome event rate of 9% per year based on prior trials.^8-10^ This was revised to 10% per year based on the observed aggregate primary outcome event rate in ACHIEVE.
3. Cross-over: We originally assumed 20% of the randomized participants would permanently discontinue spironolactone within the first year and that an insignificant number of participants in the placebo group would start spironolactone. The observed aggregate ACHIEVE data suggested 14.8% of participants permanently discontinued study drug (either active or placebo). While this could have meant as much as 29.6% of participants discontinue active spironolactone this seemed unlikely given the run-in phase to exclude spironolactone related side effects and the low rate of permanent discontinuation for known spironolactone related side effects. We therefore conservatively estimated that between 50% and 75% of discontinuations were in the active arm, with 75% in the first year.
4. Loss to follow-up: Because dialysis is a life sustaining therapy and movement between dialysis units typically involves the transfer of medical data from unit to another, tracking the movement of patients who require dialysis within a jurisdiction or country is typically straightforward. Although lost to follow-up is uncommon in dialysis, we expected 4% per year to receive a renal transplantation based on other trials and epidemiological data. We planned for these patients to have their analysis time censored at the time of transplantation and for the purposes of sample size, thought of as lost to follow-up due to the early right censor for the primary outcome.
5. Accrual period and follow-up: The original 3 years accrual was re-estimated based on the actual accrual rate, incorporating a vanguard phase and the effects of the global COVID-19 pandemic, as 6 years.
6. Follow-up period of 1 to 2 years after last patient randomized.

Under the above assumptions, the estimated sample size required to achieve 90% power with 2-sided alpha .05 was originally 1324 participants per group randomized (2648 randomized participants total) with an expected 700 outcome events. Under the revised assumptions, the desired power would be reached at 650 primary outcomes.

### Statistical Analysis

All primary analyses will analyze all participants in the group to which they were randomized irrespective of therapy received (ie, according to the intention-to-treat principle). Given the expected cross-over rate, we also planned sensitivity analyses for a per-protocol population that censored participants at the time of becoming nonadherent (taking less than 50% of the study medication) or discontinuing their study medication. These analyses are fully specified in a Statistical Analysis Plan finalized prior to unblinding.

We will analyze the time to first occurrence of the primary composite outcome using the cause specific Cox model accounting for the potentially significant effects of competing risk due to noncardiovascular mortality. If the effect on noncardiovascular mortality is in the opposite direction as the primary outcome, we will report the subdistribution hazard ratio for primary outcome while jointly considering the competing risk using the methods described by Fine and Gray^31^ as suggested by Varadhan et al.^32^ We will infer statistical significance if the computed 2-sided *P*-value is <.049659 (according to the interim analyses).

Subgroup analyses will be performed by including interaction terms between the subgroup and the treatment allocation in the appropriate models for the primary outcome. A priori, we selected history of heart failure (any history vs no history), sex (male vs female), dialysis vintage (1 year or less vs greater than 1 year), and history of coronary artery disease (any history vs no history) as subgroups for the primary outcome and included the rationale and hypothesized direction of effects in our statistical analysis plan in accordance with recommendations.^33^ Briefly, we hypothesized that females, patients with heart failure, newer dialysis vintage, or a history of coronary artery disease may benefit from spironolactone to a greater extent than others due to either fewer side effects/improved adherence, an observed benefit in other populations, or a greater potential to alter the natural history of the structural cardiac disease observed in kidney failure.

## Trial Registration, Ethical Approvals, and the Role of Funders

The trial was registered at clinicaltrials.gov (NCT03020303) before trial recruitment began. The trial was conducted in accordance with Good Clinical Practice guidance and the principle of the Declaration of Helsinki. Each center was required to submit the protocol to an ethics review committee or similar body (eg, institutional review board or research ethics board) and forward a copy of the signed approval to the sponsor prior to beginning recruitment. The funders had no role in the design of the trial, the collection of data, the analyses, the interpretation of results, the writing of the manuscript or the decision to publish the results.

## Progress

We consented 3689 participants from 143 centers in Australia, Brazil, Canada, China, Ecuador, India, Malaysia, Aotearoa New Zealand, the Philippines, Türkiye, the United Kingdom, and Uruguay between April 2018 and December 2024. Of these, 3565 were eligible and entered run-in. During the conduct of the trial we dealt with both the COVID-19 global pandemic and a recommendation to end the trial before accruing 650 primary outcome events.

The global COVID-19 pandemic resulted in a pause in center activation and recruitment in most sites and on-site monitoring at all sites. In the Philippines, the pandemic resulted in a reorganization of patients receiving dialysis away from centers in Manila where all Philippines ACHIEVE centers were located. ACHIEVE participants in the Philippines were almost universally sent to distant, centers not participating and not able to participate in the trial, resulting in their premature exit from the study. Outside of the Philippines, ACHIEVE participants already in run-in continued to be followed as per the protocol, which allowed remote visits and study drug resupplies. We added data on COVID-19 related hospitalizations, vaccinations, treatments, and deaths. During the pandemic we monitored noncardiovascular deaths to understand if a competing risk of noncardiovascular deaths due to COVID-19 would affect our sample size estimate, but no substantial change was required.

In December 2024, the External Safety and Efficacy Monitoring Committee met to review the interim data for an efficacy analysis. Based on 513 reported primary outcome events, the Committee unanimously recommended that ACHIEVE could commence an early, orderly close out because it had (1) answered the primary research question and (2) found no compelling safety issues. At that time, considering the trial was estimated to have less than a year of follow-up left to accrue its remaining events, the decision was made to accept the recommendation, and, with the investigators still blinded, accelerated close-out commenced in January 2025 with all final visits completed before February 28, 2025.

## Discussion

Cardiovascular morbidity and mortality remain high and a top concern of patients receiving dialysis. Aldosterone may be a significant cause of cardiovascular events in this population given its frequently elevated concentrations and its association with myocardial fibrosis, heart failure and sudden death all of which are frequent in patients receiving dialysis. Based on this and the effects of mineralocorticoid antagonism in related diseases like heart failure and chronic kidney disease, mineralocorticoid antagonism is a promising but unproven treatment to reduce cardiovascular events in patients receiving dialysis.

The ACHIEVE trial is an investigator-initiated, publicly funded, international RCT designed to determine if spironolactone, the most widely available MRA globally, reduces cardiovascular deaths and heart failure hospitalizations. ACHIEVE was stopped early after the accrual of over 500 primary composite endpoints with no identified safety concerns and demonstrated the feasibility of testing pharmacologic therapies in this understudied population.^34-36^

Global RCTs of relatively uncommon diseases funded by public and charitable grants are rarely easy to conduct. Completing such a trial during a global pandemic created additional struggles. ACHIEVE was designed before the pandemic to be flexible in terms of recruitment and follow-up methods to ensure it could be performed at any dialysis unit regardless of the unit’s organization, clinical process and local resources. This design made it possible to conduct ACHIEVE with its already recruited participants throughout the pandemic. Center activation and recruitment of participants amongst activated centers was substantially slowed and resulted in prolonging the study. In addition, the pandemic created unforeseen challenges including the staffing and oversight of some centers and rapid changes in costs of drug procurement, bottling, labeling and shipping. Despite the numerous challenges faced, ACHIEVE demonstrates the ability to conduct global, investigator-initiated, trials funded by public funding agencies and charitable organizations.

Even if spironolactone reduces cardiovascular morbidity and mortality in patients with kidney failure, their risk will remain high. Further strategies to improve outcomes will be needed. ACHIEVE demonstrates it is possible for investigator-initiated, international, publicly funded RCTs to significantly contribute to our understanding of treating patients with kidney failure.

## Supplemental Material

sj-docx-1-cjk-10.1177_20543581251348187 – Supplemental material for The Aldosterone Blockade for Health Improvement Evaluation in End-Stage Renal Disease (ACHIEVE) Trial: Rationale and Clinical Research Protocol Supplemental material, sj-docx-1-cjk-10.1177_20543581251348187 for The Aldosterone Blockade for Health Improvement Evaluation in End-Stage Renal Disease (ACHIEVE) Trial: Rationale and Clinical Research Protocol by Michael Walsh, David Collister, Martin Gallagher, Patrick B. Mark, Janak R. de Zoysa, Jessica Tyrwhitt, Karthik Tennankore, Laura Sola, Gilmar Reis, Denis Xavier, Russell Villanueva, Wen J. Liu, Camilo Félix, Li Zuo, Mustafa Arici, Vivekanand Jha, Ron Wald, Amanda Y. Wang, Atiya R. Faruqui, Fei Yuan, Shun Fu Lee, Alena Kuptsova, Courtney Christou and P. J. Devereaux in Canadian Journal of Kidney Health and Disease
