# Supplementary material for: The Aldosterone Blockade for Health Improvement Evaluation in End-Stage Renal Disease (ACHIEVE) Trial: Rationale and Clinical Research Protocol
Source: Can J Kidney Health Dis. 2025 Jun 3;12:20543581251348187. doi: 10.1177/20543581251348187 (PMC12134517; doi:10.1177/20543581251348187)
Supplement: sj-docx-1-cjk-10.1177_20543581251348187 – Supplemental material for The Aldosterone Blockade for Health Improvement Evaluation in End-Stage Renal Disease (ACHIEVE) Trial: Rationale and Clinical Research Protocol [file sj-docx-1-cjk-10.1177_20543581251348187.docx]

# Supplemental Material

## Appendix 1. ACHIEVE Investigators and Committees

**Steering Committee:** P.J. Devereaux (Chair), M. Walsh (Global Principal Investigator), M. Arici, J.R. de Zoysa, C. Félix, M. Gallagher, V. Jha, W.J. Liu, P.B. Mark, G. Reis, L. Sola, K. Tennankore, R. Villanueva, R. Wald, A. Wang, D. Xavier, L. Zuo

**Event Review and Adjudication Committee:** J.F.E. Mann (Co-Chair), A. Smyth (Co-Chair), D. Collister, T. Ferreira, M. Junek, D. Massicotte-Azarniouch, P. Roshanov, E. Wu, N. Ye

**External Safety and Efficacy Monitoring Committee:** P. Parfrey (Chair), M. Pfeffer, S. Pocock, M. Tonelli

**United Kingdom Independent Trial Steering Committee:** D. Jayne, A Webster, S. Mitra, A. Al-Gabry

**Project Office Study Staff:** J. Tyrwhitt, C. Christou, A. Kuptsova, K. McKay, C. Hardy, C. Lapensee, J. Wilkinson, K. Pohl, K. Ou-Yang

**ICT Support, Statisticians & Programmers:** H. Wilton, K. Commanda, M. Smart, F. Yuan, J. Liu, S. Lee, P. Gao, K. Balasubramanian, M. Molec, E. Dai, D. Wijesena, M. Renters, N. Jethoo

**National Leaders’ Office Staff for Coordination and Monitoring by Country:** **TGI Australia:** M. Gallagher, A.Y. Wang, Y. Mehta, V. Gregory, J. Ramos, J. Chakraborty, A. Malik, P. Ghosh, H. Man, S. Coggan **Brazil:** G. Reis, R. Oliveira, **Canada:** K. Tennankore, A. Mazzetti, **TGI China:** L. Zuo, A.Y. Wang, I. Yue, J. Zhou, **Ecuador:** C. Félix, M. López-Flecher **India:** D. Xavier, V. Jha, A.R. Faruqui, F. Xavier, P. Girish, R. Giriraju, S. Subbin, J. Rao, **Malaysia:** W.J. Liu, **Aotearoa** **New Zealand:** J.R. de Zoysa, M. Baker, L. Rive, **Philippines:** R. Villanueva, **Turkey:** M. Arici, **United Kingdom:** P.B. Mark, L. Gillespie, E. Moody, S. Fairley, S. Mason, D. Taggart, P. Surtees, E. Douglas, S. Carmichael **Uruguay:** L. Sola

**Site Principal and Co-Investigators (*)** number of participants randomized

**Australia (95*):** M. Gallagher, P. Branley, S. Campbell, V. Campbell, M. Cheetham, Y. Cho, A. Flavell, C. Foote, R. Francis, M. Geot Wong, N. Gray, J. Ha, P. Hollett, M. Jardine, J. Kent, J.P. Killen, R. Krishnasamy, C. Lai, V. Levidiotis, F. Magid, K. Mahadevan, L. McMullen, D. Mudge, E. Noble, D. Palammuthusingam, E. Pedagogos, K. Polkinghorne, S. Putrino, M. Razavian, A. Ritchie, S. Sen, Y. Shen, A. Siriwardana, G. Talaulikar, C. VanEps, G. Walters, A.Y. Wang, J. Waugh, M. Wolley, R. Wyndham

**Brazil (466*):** A. Alvarenga, V. Antunes, M. Augusto Cray da Costa, M. Bacci, G. Baroni, L. Belinaso, I. Boehm, V. Calice da Silva, S. Cantini, L. Cardon de Oliveira, M. Carlos Riella, S. Castro, C.P. Correia, P. da Cruz Queiroz, P. da Silva Correia, A. de Padua Lanna, L.V. de Souza, G. de Souza Pantano, T. Dias, S. Dias Rodrigues, M. E. Hernandes, T. Ferreira Dias, L. Filogonio, M. Franca Ferreira, R. Franco, R. Furtado de Carvalho, J. Goldani, V.A. Hamamotto Sato, M. Hanauer, V. Hipólito, C. Kenji Makashima, L. Mafra, G. Mandelli, G. Marcal, C. Marmanillo, L. Martin, L. Melo, S. Miquelanti Terto, G. Moreno Gordon, P. Neves, A. Nicolodi, G. Paula, A. Pedros dos Santos, G. Pereira Junior, A. Picolli, P. Queiroz, H. Rebello Narcizo, C. Richter, H.G. Rogognete, S. Santana, A.F.M. Schuinski, G. Sevignani, R.F. Silva, F. Thome, M.J. Tostes, M.E. Yokota, S. Zimmermann

**Canada (604*):** K. Tennankore, M. Agharazii, W. Beaubien-Souligny, A. Bello, D. Blum, J. Bouchard, S. Brimble, V. Casilla, J. Conley, S. DeSerres, S. Desmeules, B. Forzley, L. Girard, S. Gonzalez, Z. Harel, J.P. Harmon, B. Hemmelgarn, S. Hiremath, R. Holden, A. Jamal, M. Kiaii, B. Kiberd, A. Kitchlu, J. Lafrance, D. Lagrotteria, L. Laurin, A. Levin, S. Lyle, F. Madore, B. Manns, M. Michaud, A. Molnar, N. Muirhead, A. Nadeau-Fredette, G. Nesrallah, S. Netherton, A. Nistico, G. Ouellet, N. Pannu, J. Perl, V. Pichette, A. Priario, L. Pyne, C. Rabbat, J. Rioux, J. Sasal, N. Scott-Douglas, L. Sola, S. Soroka, R. Suri, S. Thanamayooran, B. Thomson, M. Vallee, R. Wald, M. Weir, D. Zimmerman

**China (208*):** L. Zuo, L. Chen, M. Chen, Y. Chen, F. Gao, L. Gao, C. Guo, Y. Guo, Y. Jing, R. Li, B. Liu, Z. Liu, J. Pang, Y. Shen, J. Shi, Y. Sun, J. Tian, C. Wang, S. Wei, Y. Xu, C. Yu, G. Zhang, J. Zhang, Q. Zhang, X. Zhang, Z. Zhang, X. Zhou

**Ecuador (124*):** D. Almeida, S. Carrasco, I. Martinez, M. Sanchez, S. Valarezo, V. Vizcaino

**India (312*):**  F. Ahmad Abdul Irfan Ansari, A. Almeida, Y.J. Anupama, S. Balamurugan, I. Basheer, R. Bhat, T. Dineshkumar, R. Divivedi, P. Gaggar, N. Gopalakrishnan, K. Ismal, V. Jeeva, A. John, J. Kamath, M. Kulkarni, V. Kumar, A. Magal, M. Manish, R. Manorajan, S. Manuael, L. Marisiddappa, C. Netharakere, A. Oomman, R. Padmaraj, A. Parekha, P. Pragna, D. Premkumar, P.V. Rahate, G. Raja, C. Rakesh Durai, S.M. Rao, M. Ravitej, M. Sahay, R. Sakthirajan, P. Shenoy, D.G. Shiva Kumar, D. Shivakumar, R. Sirsat, D. Sree Bhushan Raju, R. Sreedhara, S. Sulthan Alavudeen, G.A. Suresh, A. Venkatesh, R. Venkatramanan, P. Visweswar Reddy, K.G. Wadhai

**Malaysia (310*):** W.J. Liu, A. Abdul Aziz, Z. Abdul Aziz, M. Abdul Wahab, M. Ahmad, N. Ahmad, N. Ahmad Miswan, D. Arimuthu, A. Bahtar, S. Bavanandam, J. Cheng, M. Cheng, Y. Cheng, J. Choe, L. Ee, M. Fu, K. Lam, S. Lee, Y. Lee, Y. Liew, S. Mazlan, F. Mohamad Nor, M. Mohd Din, I. Muhammad Amin, A. Murugiah, L. Mushahar, N. Mustapa Albakari, M. Nadesan, M. Narthan, N. Nordin, Y. Nurul Ain, Y. Ong, R. Ramli, S. Shah Firdaus Khan, H. Shuhaimi, G. Siew, M. Tan, K. Teng, A. Usamah, W. Wan Md Adnan, H. Wan Mohamad, I. Wong, R. Yahya, S. Yee, H. Zaiha, N. Zainal Abidin

**Aotearoa New Zealand (101*):** J.R. de Zoysa, H. Allawati, J. Devathasan, M. Hassan, A. McNally, A. Mullan, J. O' Riordan, N. Panlilio, T. Putt, K. Rabindranath, S. Sapsford, R. Walker

**Philippines (9*):** R. Villanueva, R. Tan

**Turkey (14*):** M. Arici, B. Görҫin, S. Güvenir Özkurt, G. Haberal, T. Olgac Sarikulak, R. Tektaş

**United Kingdom (241*):** P.B. Mark, J.J.V. McMurray, T.F. Hiemstra, P.A. Kalra, P.R. Kalra, A.P. Maxwell, D.C. Wheeler, A. Abbas, B. Ajayi, J. Allen, C. Bebb, S. Bell, L. Bisset, K. Bramham, J. Burton, R. Chinnadurai, C. Chukwu, L. Clark, T. Connor, I. Dasgupta, T. Dasgupta, T. Doulton, C. Farmer, C. Ferro, A. Forbes, C. Goldsmith, C. Goldsmith, J. Graham, A. Hameed, C. Hill, N. Hoye, F. Ibrahim Lutfi, M. Jesky, A. Karim, D. Keenan, M. Lambie, N. Leonard, M. Leung, K. MacConaill, A. Masengu, K. McCafferty, R. McCrory, E. McQuarrie, A. Menon, S. Methven, S. Mohir, N. Morgan, T. Navodhi Somasinghe, B. Pandya, P. Papadopoulou, R. Patel, K. Ping Ng, A. Power, R. Pyart, A. Randhay, A. Rao, N. Selby, E. Sharples, T. Shipley, P. Smith, J. Smyth, J. Storrar, S. Stoumpos, M. Sullivan, P. Swift, B. Tan, P. Thing, M. Wahba, A. Woodman

**Uruguay (54*):** S. Sola, C. Baccino, J.C. Diaz, S. Gonzalez, R. Menoni, D. Miller

## Appendix 2. Outcome Definitions

**Cardiovascular death:** deaths will be assumed to be cardiovascular in nature unless a non-cardiovascular cause can be clearly identified (e.g. malignancy, withdrawal of dialysis, suicide, accidental death). Death will be considered non-cardiovascular only if an unequivocal and documented non-cardiovascular cause can be established. Causes of cardiovascular death will include, myocardial infarction, heart failure, pulmonary edema, cardiogenic shock, sudden death (witnessed or unwitnessed), stroke, death following a cardiovascular procedure such as coronary artery bypass, percutaneous coronary interventions, and valvular procedures and other cardiovascular causes. Deaths following non-cardiovascular events (e.g. withdrawal of dialysis and non-cardiovascular procedures) will still be considered cardiovascular deaths if a non-fatal cardiovascular event occurred within 30-days of death. For example, the withdrawal of dialysis within 30 days after a stroke will be considered a cardiovascular death. Similarly, death that occur within 30 days after a non-cardiac surgery that was complicated by a myocardial infarction would be considered a cardiovascular death. We will utilize a modified verbal autopsy for deaths that occur out of hospital for which there are no medical records.

**Non-cardiovascular death:** deaths will be considered non-cardiovascular only when an unequivocal non-cardiovascular cause can be established and documented. Non-cardiovascular causes include but are not limited to: withdrawal of dialysis unless withdrawal precipitated by a cardiovascular event (e.g. refractory angina or cardiogenic dyspnea), malignancy, chronic pulmonary disease (excluding cardiogenic pulmonary edema), infections, hepatobiliary disease, gastrointestinal disease, venous thromboembolic disease, accidents/trauma, and metabolic disorders (e.g. hyperkalemia induced cardiac arrest).

**Cause specific death categories:** Cardiac deaths are defined as deaths due to heart failure/pulmonary edema, cardiogenic shock, or sudden death/arrhythmia. Vascular deaths are defined as deaths due to MI, after invasive CV intervention, stroke withdrawal of dialysis, or other cardiovascular causes. Non-cardiovascular deaths are all deaths that are neither cardiac nor vascular (i.e. non-cardiovascular causes).

**Hospitalization for heart failure**: hospitalization for heart failure requires an unplanned admission to a hospital with signs or symptoms of heart failure, and unplanned mechanical fluid removal (e.g. dialysis or ultrafiltration occurring at a time or in a place that is different from their usual dialysis).

Signs and symptoms of heart failure are dyspnea without an obvious alternative cause (e.g. active pulmonary infection or venous thromboembolism) with at least one of the following:

- Bilateral basilar rales on physical exam
- Interstitial edema on chest X-ray or other radiological investigation
- Apical vascular redistribution/increased upper pulmonary vessel diameter on chest X-ray
- Elevated left ventricular end-diastolic pressure or pulmonary capillary wedge pressure by Swan-Ganz catheter
- Lung ultrasound demonstrating interstitial edema

When multiple potential causes for dyspnea exist, symptomatic improvement attributed to mechanical fluid removal is considered evidence of heart failure if there is at least one other sign of heart failure.

**Myocardial infarction:** myocardial infarctions may be typical or post cardiac procedure. *Typical myocardial infarctions* require biochemical evidence and concomitantly either ECG changes, cardiac imaging changes, or symptoms indicative of a myocardial infarction.

Biochemical evidence of a myocardial infarction includes any of the following:

- Changes in troponin T or troponin I with a peak measured value that must exceed the necrosis limit for the local assay
- Changes in CK-MB with a peak measured value that is at least 2 times the upper limit of normal of the local assay

ECG changes indicative of a myocardial infarction include any of the following:

- New significant Q waves (or R waves in V1-V2) in two or more contiguous leads in the absence of confounding conduction abnormalities
- Dynamic ST-segment elevation of at least 1 mm in two or more contiguous leads
- Dynamic ST-segment depression of at least 1 mm in two or more contiguous leads
- Dynamic T-wave changes
- Development of new left bundle branch block

Cardiac imaging indicative of a myocardial infarction include:

- New or presumed new wall motion abnormalities

Symptoms of a myocardial infarction include:

- Angina/chest discomfort
- Dyspnea
- Cardiac arrest
- Ventricular arrhythmia

*Cardiac procedure related myocardial infarctions* are myocardial infarctions that occur within 72 hours of cardiac or non-cardiac procedure. The criteria for these are different as cardiac procedures (percuraneous interventions or cardiac surgery) may directly result in the release of cardiac enzymes and alter the ECG and post-operative analgesia (cardiac surgery and non-cardiac surgery) reduces the sensitivity of ischemic pain and dyspnea.

Post-percutaneous intervention myocardial infarction: a change in troponin to at least 3x the necrosis limit, or CK-MB to 3 x the upper limit of normal or persistent new pathological Q waves or a new or presumed new wall motion abnormality on cardiac imaging.

Post-cardiac surgery (non-valvular surgery): a change in troponin to at least 5x the necrosis limit, or CK-MB to at least 5x the upper limit of normal, with new pathological Q-waves on ECG.

Post-cardiac surgery (valvular surgery): a change in troponin to at least 7x the necrosis limit, or CK-MB to at least 7x the upper limit of normal, with new pathological Q-waves on ECG.

**New onset atrial fibrillation:** atrial fibrillation documented on electrocardiogram and not previously documented.

**Severe Hyperkalemia:** severe hyperkalemia will be defined as any temporary or permanent discontinuation due to hyperkalemia, any hospitalization due to hyperkalemia or any follow-up potassium recorded as >6.5 mmol/L.

**Stroke:** a focal neurological deficit, resulting from a vascular cause involving the central nervous system, that is not reversible within 24 hours and which is not due to a readily identifiable alternative cause (e.g. brain malignancy, trauma).
